# Trends and Incidence of Hearing Implant Utilization in Italy: A Population-Based Study

**DOI:** 10.3390/audiolres15060175

**Published:** 2025-12-14

**Authors:** Enrico Ciminello, Domenico Cuda, Francesca Forli, Anna Rita Fetoni, Stefano Berrettini, Eugenio Mattei, Tiziana Falcone, Adriano Cuccu, Paola Ciccarelli, Stefania Ceccarelli, Marina Torre

**Affiliations:** 1 Italian Implantable Prostheses Registry, Italian National Institute of Health, 00161 Rome, Italypaola.ciccarelli@iss.it (P.C.); stefania.ceccarelli@iss.it (S.C.); marina.torre@iss.it (M.T.); 2 Department of Otolaryngology, AUSL Piacenza, 29121 Piacenza, Italy; d.cuda@ausl.pc.it; 3 Otolaryngology, Audiology, and Phoniatrics Unit, University of Pisa, 56124 Pisa, Italy; francesca.forli@unipi.it (F.F.); stefano.berrettini@unipi.it (S.B.); 4 Department of Neuroscience, Reproductive Sciences and Dentistry, University of Naples Federico II, 80131 Naples, Italy; annarita.fetoni@unina.it; 5 Department of Cardiovascular, Endocrine-Metabolic Diseases and Aging, Italian National Institute of Health, 00161 Rome, Italy; 6 Department of Statistical Science, “La Sapienza” University of Rome, 00185 Rome, Italy

**Keywords:** hearing loss, cochlear implant, hearing devices, routinely collected health data, registries, epidemiological monitoring

## Abstract

**Background/Objectives:** Cochlear implants (CIs) and other implantable hearing devices are crucial to treat hearing loss. The aim of this study was to analyze the temporal trends of implantation for hearing devices in Italy between 2001 and 2023, with stratification by age. **Methods:** This population-based study explored Hospital Discharge Records and used codes from the International Classification of Diseases, 9th revision—Clinical Modification (ICD9-CM) to identify cochlear and non-cochlear implants. Patients were partitioned into six age classes: <1, 1–2, 3–17, 18–65, 66–80, and >80; and time series for counts and incidence rates (IRs) per 1,000,000 inhabitants with confidence intervals (CI_95%_) were explored overall and by age class. Trends were assessed by incidence rate ratio and Cox–Stuart test with a significance threshold for *p*-values at 0.05. **Results:** 22,850 (83.6%) records for cochlear and 4476 (16.4%) for non-cochlear implants were extracted. Cochlear implants volume shifted from 537 procedures in 2001 to 1595 in 2023 (*p* < 0.01), while IR increased (*p* < 0.01) from 9.4 (CI_95%_: 9.7, 10.3) in 2001 to 27 (CI_95%_: 25.7, 28.4) in 2023. The volumes of implanted CIs increased in children and adults. Volumes for non-cochlear implants increased between 2001 and 2010, from 62 to 254, and remained stable afterwards. IR shifted from 1.1 (CI_95%_: 0.8, 1.4) in 2001 to 4.1 (CI_95%_: 3.6, 4.7) in 2023. **Conclusions:** Those trends highlight the importance of monitoring efficacy and safety of hearing devices, and the establishment of the Italian Implantable Hearing Device Registry at the Italian National Institute of Health is a first step in such a direction.

## 1. Introduction

Over the past few decades, cochlear implants (CIs) and other implantable hearing devices have become increasingly common in the treatment of hearing loss, driven by technological advancements and the broadening of clinical indications. In that period, some clinical events likely shaped implantation volumes in Italy, including the broadening of CI candidacy in national guidelines, the progressive implementation of universal newborn hearing screening, and the temporary suspension of elective surgeries during the COVID-19 pandemic. Initially reserved for patients with total bilateral deafness, CIs are now indicated for individuals with residual hearing, bimodal and bilateral treatments, Single-Sided Deafness (SSD), and for both very young children (under the age of one) and elderly patients [1,2,3,4,5]. The success of CIs is unmatched among neuroprosthetic treatments because of developed features like improved speech processing strategies, optimized electrode designs, reduced energy consumption, and the miniaturization of external processors. Definitely, those factors have significantly enhanced both clinical and everyday-life outcomes, as well as patients’ acceptance. At the same time, the use of non-cochlear implantable hearing devices (such as middle ear prostheses and bone-anchored hearing aids) has also expanded, particularly since the 1980s. The usage of some devices reached a peak around 2010, but later decreased due to management difficulties, costs, and complications. On the other hand, other tools, like bone-anchored hearing devices, have continued to grow in popularity, especially with the introduction of active percutaneous models [6]. Despite the widespread use of these devices, there is a significant lack of systematic and updated data at the population level targeting the volumes of implantation and population incidence in Italy. This information is essential to understand clinical trends, identify inequalities in access to care, and guide effective healthcare planning. Indeed, some population-level and registry-based studies have been carried out in other European and extra-European countries, providing interesting results when interpreting temporal trends [7,8,9,10,11].

The aim of this study is to analyze the volumes of implantation of CIs and other implantable hearing devices in Italy between 2001 and 2023, with a focus on temporal trends overall and with stratification by age. The goal is to provide a comprehensive overview of national volumes and derive valuable insights for clinicians, health administrators, and policymakers.

## 2. Materials and Methods

This observational, population-based study was carried out according to the Strengthening the Reporting of Observational Studies in Epidemiology (STROBE) guidelines provided by the Enhancing the Quality and Transparency of Health Research (EQUATOR) Network [12]. Finally, the study passed all checks of the Reporting of studies Conducted using Observational Routinely-collected health Data (RECORD) Statement [13].

### 2.1. Data Source

The Italian Ministry of Health collects data about every hospital discharge occurring in Italy every year and feeds the Italian Hospital Discharge Record (HDR) database with it. The estimated completeness reached with the records included in such a database with respect to the overall number of hospital admissions occurring in Italy reaches 99% [14]. Every year, the Ministry of Health transmits a subset of variables of the complete HDRs, useful for epidemiological studies, to the Italian National Institute of Health (Istituto Superiore di Sanità—ISS). HDRs provide administrative, demographic, and clinical information, including a maximum of one main and ten secondary procedures performed during the hospital stay, and a maximum of one main and five secondary related diagnoses, reported by using the codes in the International Classification of Diseases, 9th revision—Clinical Modification (ICD9-CM).

### 2.2. Study Design

First, four experienced medical doctors and university professors (DC, ARF, FF, and SB) blind-selected ICD9-CM codes of interest from 4460 procedure codes of the 2007 Italian version of the ICD9-CM manual, based on their knowledge and expertise. On full blind consensus, ICD9-CM codes were included in the list of the codes of interest; on partial consensus (3/4 among the experts), the inclusion of the code was discussed among experts until a final complete consensus was reached; if less than three of the experts considered a code eligible, then the code was excluded. Second, clinical experts agreed on mapping codes of interest onto two implant categories: “Cochlear” and “Non-cochlear”, where the “Non-cochlear” category covered all types of other hearing implantable devices, middle ear implants, and bone-anchored hearing devices included. Last, starting from 239,560,403 HDRs collected in Italy in the years between 2001 and 2023, the records including the ICD9-CM codes of interest reported in Table 1 among the main or secondary procedure fields were extracted and labeled as related to the implant of either a non-cochlear or a cochlear device. A further filtering step was performed, ensuring that the sex and age of the patient were correctly reported. Ambiguous records, reporting the implantation of both cochlear and non-cochlear devices, were excluded.

### 2.3. Measures

Time series of counts and incidence rates (IRs) × 1,000,000 inhabitants, based on census data on the population provided by the Italian National Statistical Institute (https://esploradati.istat.it/databrowser/#/, accessed on 5 December 2025), were explored. A stratification by sex and age, independently and combined, was performed. Those were the only relevant individual patients’ features available in the database for a thorough exploration of the implantation patterns. Age was grouped in six classes according to the following criteria: <1 year of age; 1–2 years; 3–17 years; 18–65 years; 65–80 years; and over 80 years of age. This partition allows for assessing the trend of the procedure for both pediatric patients, given the implications a hearing device may have in relation to brain plasticity, and adults, with a focus on elderly patients, highlighting how they have received an indication for the procedure over the years. Moreover, given the changes in age distribution in the Italian population over the time period under analysis, such age classes were used to compute age-adjusted IRs (AAIRs), in order to provide comparable outcomes between years. Finally, a cohort study by year of birth was conducted to check for possible shifting in the age of implants in the youngest patient population, taking into account IRs of cochlear devices implanted by the fourth year of age, for all cohorts in which 4 observation times were available (2001–2019). In such an analysis, IRs are computed on newborns in the cohort for each year.

### 2.4. Statistical Analysis

Age was reported in terms of mean (standard deviation) and counts in terms of integers (percentage). Differences in age distribution between kinds of device (cochlear vs. non-cochlear) along age classes were tested via a chi-squared test. IRs and AAIRs were estimated via a Poisson model and reported along with 95% confidence intervals (CI_95%_), while comparisons between years were expressed in terms of incidence rate ratios (IRRs) with CI_95%_. AAIRs were computed using the 2001 Italian population as a reference. The overall trends of time series were assessed as increasing or decreasing via a Cox–Stuart test, to ensure an overall evaluation of the time series dynamics and check for possible random fluctuations. The shifting in distributions of age of implant across the years in the cohort study was tested by the chi-squared test. The threshold for *p*-values to determine statistical significance was fixed at 0.05. The statistical analysis was performed by using the software R version 4.4.2 (31 October 2024 ucrt)—“Pile of Leaves”.

## 3. Results

**Figure 1:**
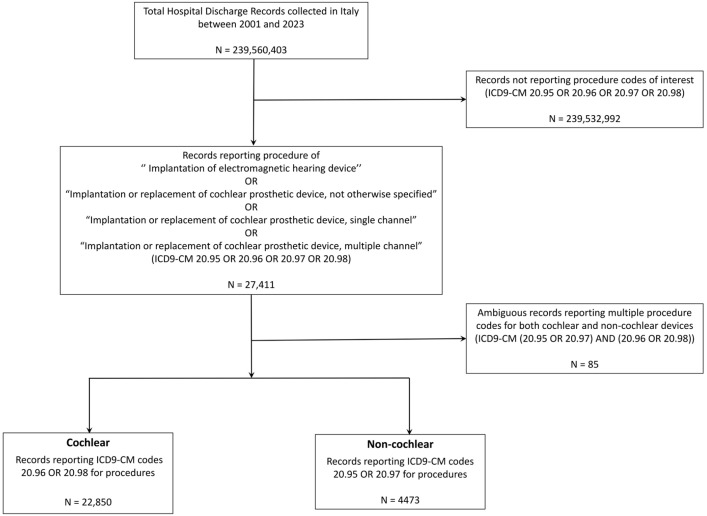
Flowchart of the data extraction process from the HDR Italian database.

The selected ICD9-CM codes and their mapping onto the two defined categories are reported in Table 1. The extraction process, carried out according to such codes, resulted in 27,326 records of hospital admission for hearing device implantation, divided into 22,850 (83.6%) cochlear and 4476 (16.4%) non-cochlear implants (Figure 1).

Patients undergoing cochlear device implantation were significantly (*p* < 0.01) younger with respect to patients undergoing non-cochlear implantation, with 39% and 12.8% of the patients under the age of 18, respectively. The population characteristics by sex and age class are summarized in Table 2.

### 3.1. Overview

**Figure 2:**
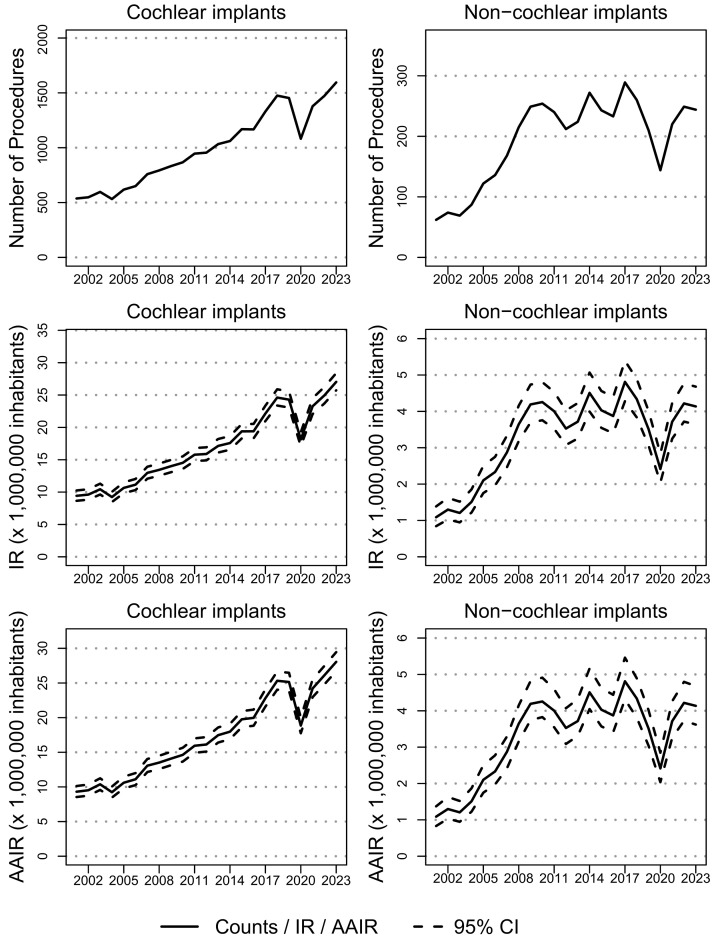
Counts, incidence rates (IRs), and age-adjusted incidence rates (AAIRs) for implanted cochlear and non-cochlear devices in Italy between 2001 and 2023.

**Figure 3:**
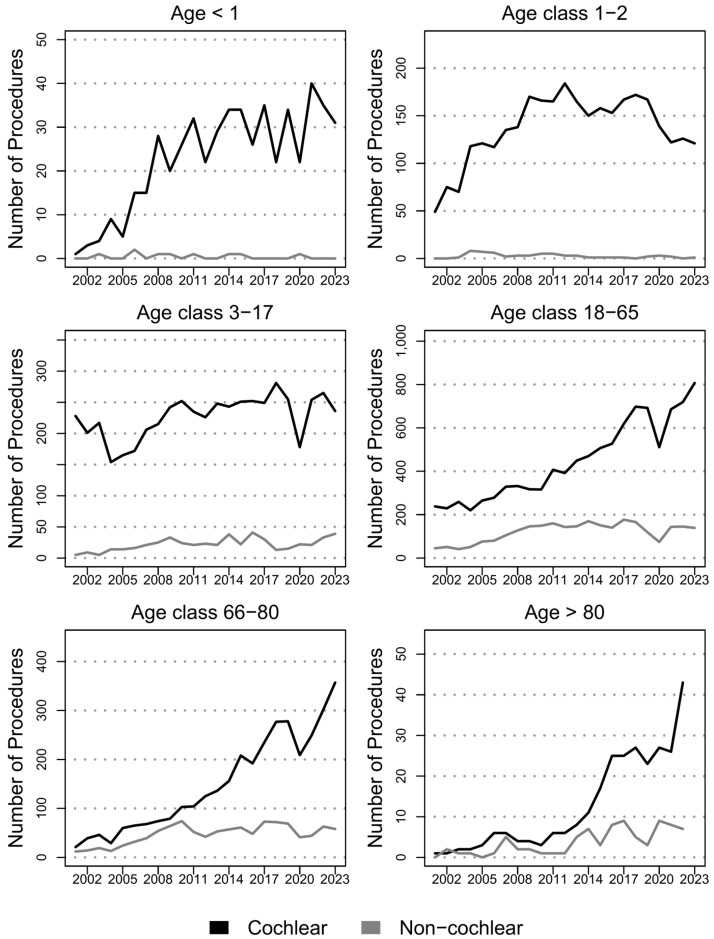
Counts for implanted cochlear and non-cochlear devices in Italy between 2001 and 2023 by age class.

The number of implanted hearing device increased from 599 in 2001 to 1839 in 2023 (*p* < 0.01) and the same is observed when looking at IR, which trebled (*p* < 0.01) form 10.5 (CI_95%_: 9.7, 11.4) implanted devices per 1,000,000 inhabitants in 2001 to 31.2 (CI_95%_: 29.8, 32.6) in 2023, as confirmed by the IRR equal to 3 (CI_95%_: 2.7, 3.3). The exploration of AAIRs confirmed the findings reported without adjusting for age, with similar figures, and provided information. Time series for counts, IRs, and AAIRs for implanted devices by type, sex, and age class are reported in Figure 2. The results of age class stratification are reported in terms of counts and IRs in Figure 3 and Figure S1, respectively. All figures for trends by age class and overall are detailed in Supplementary Materials, Tables S1–S3 (SupplementaryMaterial_TableS1.xlsx) with CI_95%_, IRRs, and statistical testing results. Moreover, a stratification by sex is only added in the Supplementary Material as no relevant differences by sex were detected.

### 3.2. Cochlear Implants

Cochlear implants volume almost tripled, from 537 procedures in 2001 to 1595 in 2023 (*p* < 0.01), while IR increased (*p* < 0.01) from 9.4 (CI_95%_: 9.7, 10.3) per 1,000,000 inhabitants in 2001 to 27 (CI_95%_: 25.7, 28.4) in 2023, with IRR equal to 2.9 (CI_95%_: 2.6, 3.2). Looking at age class stratification, the volumes of implanted cochlear devices increased in all age classes, except for patients between 1 and 2 years of age (*p* = 0.11), for which a spike between 2001 (49 procedures) and 2009 (170 procedures) and a drop until 2023 (121 procedures) were observed. Despite its shape, depending on the small number of cases, the trend for age class < 1 year was significantly increasing (*p* < 0.01) over the 23 observed years.

On the contrary, at age class 3–17, a drop in the number of cochlear procedures was observed between 2001 and 2004, shifting from 218 to 154 implanted devices, with an increasing trend (*p* < 0.05) in the following years until reaching 236 procedures in 2023. For patients over 80 years of age, the number of implanted devices starts from 0 in 2001 and reaches 43 units in 2023, with a spike after 2014. The highest IR is observed in age class 1–2, with 46.6 (CI_95%_: 34.9, 61.1) devices per 1,000,000 inhabitants in 2001, increasing up to 148.4 (CI_95%_: 123.7, 176.6) in 2023, with IRR equal to 3.2 (CI_95%_: 2.3, 4.4). The highest growth in IR is observed in the population under 1 year of age, with an IRR equal to 42 (CI_95%_: 5.7, 307.6), due to an IR equal to 1.9 (CI_95%_: 0.2, 8.7) procedures per 1,000,000 inhabitants in 2001, increasing to 78.7 (CI_95%_: 54.5, 110.2) in 2023. The only age class for which the growth was not significant when comparing 2001 and 2023 is the age class 3–17, with IR shifting from 27.5 (CI_95%_: 24.1, 31.2) to 30 (CI_95%_: 26.4, 34) with IRR equal to 1.1 (CI_95%_: 0.9, 1.3). It is interesting to notice that this results from a drop in IR until 2004, with a value equal to 18.7 (CI_95%_: 15.9, 21.9), followed by a significant increase until 2023 (*p* < 0.05). Last, IR for population over 80 years of age significantly (*p* < 0.01) increased from 0 (CI_95%_: 0, 1.2) procedures per 1,000,000 inhabitants in 2001 to 10.6 (CI_95%_: 7.8, 14.2) in 2023, with IRR equal to 22.5 (CI_95%_: 3.1, 163.3).

**Figure 4:**
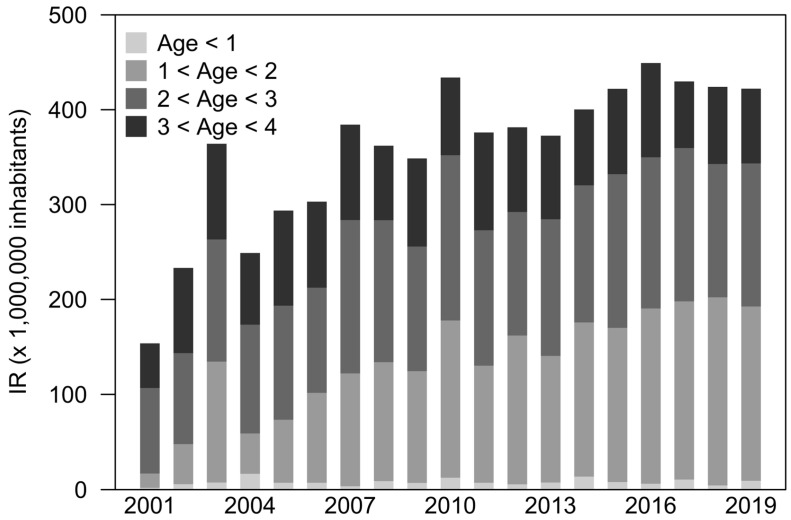
Age of treatment in patients up to 4 years of age for the cohort by year of birth, with at least 4 years of observation (2001–2019).

The cohort study in the youngest (up to 4 years of age) showed a significant (*p* < 0.01) shift in age of implant with an increasing tendency to treat patients between the first and second year of age between 2001 and 2010, and a substantially constant tendency in treating patients over the age of two. Moreover, Figure 4 shows an isolated spike in the quota of patients treated under the age of two in 2003, followed by a decrease in 2004, similar to the levels observed in 2002. The cohort study in the youngest (up to 4 years of age) showed a significant (*p* < 0.01) shift in age of implant with an increasing tendency to treat patients between the first and second year of age between 2001 and 2010, and a substantially constant tendency in treating patients over the age of two.

### 3.3. Non-Cochlear Implants

Non-cochlear implant volume, after an increase (*p* < 0.01) until 2010, remained substantially stable in the last decade. IR had a spike until 2010, passing from 1.1 (CI_95%_: 0.8, 1.4) procedures per 1,000,000 inhabitants to 4.3 (CI_95%_: 3.8, 4.8) and remained stable until 2023 (*p* = 0.11), with a value equal to 4.1 (CI_95%_: 3.6, 4.7). The number of implants remained stable in five out of the six considered age classes, with a significant increase only observed in patients over 80 years of age, shifting from zero procedures in 2021 to seven procedures in 2023. IRs showed a significant increase (*p* < 0.05) in age classes 18–65 and over 80. The highest increase in IR is observed in age class 3–17 with IRR equal to 8.2 (CI_95%_: 3.2, 20.9), spiking from 0.6 (CI_95%_: 0.2, 1.3) implanted devices per 1,000,000 inhabitants in 2001 to 3.9 (CI_95%_: 2.7, 5.4) in 2009 and remaining substantially stable (*p* = 0.27) in until 2023, with a value equal to 5 (CI_95%_: 3.6, 6.7). Also, for non-cochlear implants, IR was equal to 0 (CI_95%_: 0, 1.2) in the population over 80 in 2001, but increased up to 1.7 (CI_95%_: 0.8, 3.4) implanted devices per 1,000,000 inhabitants in 2023, with IRR equal to 1.9 (CI_95%_: 0.4, 9.3).

## 4. Discussion

The aim of this study was to explore the trends of volumes and IRs of hearing device implantation in Italy between 2001 and 2023, with a focus on the changes in the standard practice of hearing loss management over more than two decades. Overall, a slightly higher proportion of female patients was observed, with 11,698 (51.5%) cases, while the 13,012 (47.6%) patients between 18 and 65 represented the largest age class. Patients undergoing cochlear implants were younger on average than patients undergoing non-cochlear implants, with the average ages equal to 34 (26.7) and 49.3 (21.3), respectively.

An increasing trend between 2001 and 2023 was observed for all categories of devices, from 599 yearly implanted devices to 1839 overall, with a drop in 2020 due to the postponement of elective surgeries due to the COVID-19 pandemic outbreak. The number of implants increased for adult recipients for both categories of device over the 23 years under analysis, while an increased number of implants for children was observed only for cochlear implants.

### 4.1. Cochlear Implants

Cochlear implants are considered a revolutionary progress in the surgical treatment of hearing loss, with a huge impact on the entire management of rehabilitation, which has significantly changed over the last decades. Thus, we observed a growth from 537 procedures in 2001 to 1595 in 2023 (*p* < 0.01). This result seems to be more impressive considering the almost trebled IR (*p* < 0.01), from 9.4 (CI_95%_: 9.7, 10.3) per 1,000,000 population in 2001 to 27 (CI_95%_: 25.7, 28.4) in 2023.

Comparing the overall numbers of CI recipients at the international level might be challenging, as these data at the population level are not available in most countries. The literature on trends in cochlear implant volumes in Europe and internationally is limited, and the available studies are difficult to compare with the present work. Existing reports generally suffer from lack of similarly long observation periods, often covering only a few years; non-systematic or incomplete data collection, frequently based on voluntary or partial reporting; temporal mismatch and outdated datasets, reflecting older candidacy criteria and technologies; and substantial heterogeneity in methodology, including differences in data sources, population denominators, and analytical approaches [7,8,9,10,11,15,16,17]. These factors make a direct comparison challenging. However, the effort to organize medical registries in several countries, such as France, Germany, the Netherlands, Sweden, and Switzerland, provides an important contribution in collecting information and obtaining real-life data and long-term outcomes [18,19,20]. Considering adults, IRs of CIs per million inhabitants ranged widely in 2016 across several European countries, with the highest value over 35 observed in Germany [7]. In the same year, IRs for adults increased in Belgium, Finland, Sweden, Switzerland, and the UK, and IRs for children increased in Belgium, Czech Republic, Estonia, Finland, Georgia, Germany, Romania, Slovak Republic, Spain, Sweden, Switzerland, and the Netherlands between 2010 and 2016, with 8–12 CIs per 10,000 newborns on average [7]. Based on a recent report on one year experience by the German cochlear implant registry, in 2022, 2176 primary implantations were performed [8], corresponding to 26.1 per million inhabitants, according to European official data on the German population in that same year [21]. Nonetheless, this result must be carefully interpreted, since data are collected by 63 out of 100 estimated hospitals performing CI implantation in Germany [22]. At the International level, Japan arguably provides the most thorough landscape on CI implantation trends, with 11,142 cases over more than 30 years of observation, where an IR equal to 8.5 CIs per 1 million inhabitants is reported in 2017 [9].

The increase in the use of CIs to treat adults might be associated with the scientific evidence of their efficacy, which has broadened the indications for treatment, previously reserved only for profound deafness. The spread of indication in the adults in the previous two decades, as shown in the present paper, might be related to the increased accessibility of CIs to a wider range of patients and be associated with the latest version of the Italian guidelines. Nowadays, diagnoses for which the use of CIs is recommended include severe-to-profound hearing loss (HL), asymmetrical HL, ski-slope HL, and even single-sided deafness (SSD) [4]. Nonetheless, most adult patients have no access to this technology [10,11,23], despite the increasing number of implanted devices in most Western countries. This can be explained by the limited healthcare infrastructure, the cost, the stigma, and the lack of awareness that need to be counteracted by increasing knowledge and interactions between patients and professionals.

The major changes in the demographics of CI patients over the last two decades in Italy, reported in the present article, may be associated with the changes in surgical candidacy criteria to CI, which have been broadened for both children and adults, as established with the publication of the national guidelines [4,5]. Indeed, when looking at age groups, CI recipients increased in all age classes, with a particular proportional increase in children, highlighting the trend of treating hearing loss with CIs at an earlier age. In Europe, until 2015, over 30% of the pediatric CI candidates underwent surgery before 24 months of age; however, in most Northern European centers, the age of implantation was about 12 months of age, and it was the earliest at 6–11 months in the Netherlands and Germany [17]. In Italy, almost 40% of implantations have been performed in children and teenagers (<18 years of age), and, particularly, 13.8% of children aged between 1 and 2 years of age. Indeed, the changing trend in the early implantation under 1 year of age, with IR in 2023 being 42 (CI_95%_: 5.7, 307.6) times higher than in 2001 for such age class, aligns with the most recent guidelines. Moreover, the increasing trend in CIs observed in patients up to 4 years of age (Figure 4) might be a consequence of the evidence on the beneficial effects on speech and cognitive development associated with early implantations [24]. A further relevant change might be found in the early diagnosis of deafness due to the progressive implementation of universal newborn hearing screening in Italy, which has been shown to lead to early diagnosis and treatment. This screening, which covered only about half of the Italian regions in 2008, became structured and systematic through the years, reaching full coverage in 2018 [25].

Volumes for age class 3–17 showed a decrease in the number of implanted CIs between 2001 and 2004, shifting from 218 to 154 implanted devices, followed by an increasing trend in the next years until reaching 236 procedures in 2023. This pattern might be interpreted as a delayed consequence of the warning released by the American Food and Drug Administration in 2002, which exposed the risk of meningitis following cochlear implantation [26,27].

An increasing use of CIs was also observed among the population over 80 years of age, for which the first implant was detected in 2004. Despite the number of recipients still being very limited, the volume reached 43 implants in 2023. This first approach to the use of CIs in such populations might find its rationale in the evidence of CIs being beneficial in restoring hearing function, communication abilities, quality of life, and even cognitive function in patients older than 80 years [28,29,30,31]. However, further studies need to clarify outcomes in the elderly and identify predictors of success, especially in speech performances, quality of life, and cognitive decline.

### 4.2. Non Cochlear Implants

Based on the criteria of data collection, this group of “Implantable hearing devices” is a broad and heterogeneous class of implantable devices, including any semi- or fully implantable device commercially available, including bone conduction devices (BCDs), middle ear implants (active and passive), auditory brainstem implants (ABIs), and electroacoustic stimulation (EAS). These devices represent a modern and innovative solution for the rehabilitation of different hearing losses and, following the technological advancement over the years, the eligibility criteria significantly changed for patients who do not benefit from conventional hearing aids [32]. The overall number of surgical procedures significantly increased from 1.1 (CI_95%_: 0.8, 1.4) procedures per 1,000,000 inhabitants in 2001 to 4.3 (CI_95%_: 3.8, 4.8) in 2010, and then it remained substantially stable until 2023 (*p* = 0.11), with 4.1 procedures per 1,000,000 inhabitants (CI_95%_: 3.6, 4.7). Despite these devices completing the choice of hearing solutions for deaf persons, the variability and unstable outcomes might have significantly affected the expansion of the eligibility to these procedures, especially in children and younger adults. Technological advances in prostheses, surgical innovations, and enhanced rehabilitation practices appear promising for new perspectives of application [6]. Further studies are needed to define the indication and lead to the best option among the available devices for each kind of hearing loss and ear anatomy.

This paper analyzed the landscape of hearing device implantation at the population level with a coverage close to 100% over 23 years in a single Western country with a public healthcare system, which ensures accessibility to surgery for all candidates, children, and adults. Indeed, in Italy, implantable hearing device surgeries have been fully covered by the National Health Service throughout the entire 2001–2023 period, with no out-of-pocket costs for patients. This universal coverage has remained unchanged over the years, and private insurance plays only a marginal role in the Italian Health System. For these reasons, socioeconomic factors had minimal influence on access to implantation in the investigated context.

Nonetheless, the present paper has some limitations. First, the data source from which evidence is provided is built for administrative purposes and for reimbursement of clinical service delivery. Its reliability is associated with the accuracy in filling information by health professionals and is impossible to measure without an audit performed at the national level, which would imply a major and almost undeliverable effort by the Italian National Health System. Second, device implantations are detected via mapping from the ICD9-CM coding system. A different identification of codes of interest and mapping might lead to different results. Third, recollecting information on hearing loss diagnoses from HDRs might be inaccurate. Indeed, HDRs report the hearing loss diagnosis only in the presence of an associated procedure, but often, only the type of hearing loss is recorded (by ICD9-CM coding), without specifying the underlying cause and whether the implantation of a hearing device would be useful. For this reason, an estimate of how many patients would benefit from a hearing device compared to the number of patients who actually receive it is not available. Fourth, HDRs report the occurrence of an implantation, but do not report any information on the device, on pre- or post-operative, or on surgical techniques.

The best tool to overcome such limitations would be the establishment of a National Registry for hearing devices, designed on purpose to collect crucial information for epidemiological and clinical research and produce evidence based on specific population data [33,34]. For this reason, the Italian Implantable Prostheses Registry, established by law at the Italian National Institute of Health by the Prime Ministerial Decree of 3 March 2017 (Decreto del Presidente del Consiglio dei Ministri 3 Marzo 2017) [35], includes the Italian Implantable Hearing Device Registry (Registro Italiano Dispositivi Uditivi—RIDIU) among its specific registries. At the time being, the RIDIU is in its design phase, and a panel of technical, epidemiological, and clinical experts was established to define the registry structure and collectable variables of interest. The information potentially collectable by the national registry would be key to understanding clinical trends, identifying possible inequalities in access to care, monitoring how indications and technologies evolve over time, and guiding effective healthcare planning. In particular, population-level data support evidence-based decisions on the allocation of resources, the organization of specialized implanting centers, and the development of national policies aimed at ensuring timely, equitable, and high-quality access to hearing-implant services.

## 5. Conclusions

This study provides the first comprehensive, long-term analysis of hearing device implantation trends in Italy, highlighting the evolution in clinical practice and healthcare accessibility over the last two decades.

This is the first nationwide study analyzing trends in hearing device implantation, based on HDRs. The analysis highlighted increased volumes in implantation procedures, driven primarily by the significant expansion of the use of CIs. Those accounted for the majority of surgeries, and their increased use was observed in all age groups, with a marked trend in both pediatric and elderly populations. The growth in early cochlear implantation among infants (supported by the widespread implementation of newborn hearing screening) and the increasing adoption in adults and octogenarians reflect major shifts in policies, awareness, and technological advancements. The impact of updated national guidelines has contributed to a broader and more inclusive standard of care. The use of non-cochlear devices has been limited, in particular in the youngest population, with a slight increase among the elderly. While their clinical utility remains significant in selected cases, variability in outcomes and a limited indication to treat may explain their slower diffusion.

The rising number of hearing devices implanted in Italy every year highlights the need for a well-functioning registry with high completeness, able to reach a high percentage of devices for long-term safety and efficacy assessments and monitoring. The establishment of the Italian Implantable Hearing Device Registry at the Italian National Institute of Health is a first step in this direction, and we look forward to its complete functioning.

## Figures and Tables

**Table 1:** ICD9-CM procedure codes of interest and related implants.

ICD9-CM	Description	Implant
20.95	Implantation of electromagnetic hearing device	Non-cochlear
20.97	Implantation or replacement of cochlear prosthetic device, single channel	
20.96	Implantation or replacement of cochlear prosthetic device, not otherwise specified	Cochlear
20.98	Implantation or replacement of cochlear prosthetic device, multiple channels	

**Table 2:** Characteristics of Italian population undergoing hearing device implantation between 2001 and 2023 by type of implant.

	Cochlear	Non-Cochlear	Overall
Age	34 (26.7)	49.3 (21.3)	36.5 (26.5)
Females	11,698 (51.2%)	2362 (52.8%)	14,060 (51.5%)
Males	11,152 (48.8%)	2114 (47.2%)	13,266 (48.5%)
Age class < 1	522 (2.3%)	9 (0.2%)	531 (1.9%)
Age class 1–2	3148 (13.8%)	58 (1.3%)	3206 (11.7%)
Age class 3–17	5225 (22.9%)	505 (11.3%)	5730 (21%)
Age class 18–65	10,267 (44.9%)	2745 (61.3%)	13,012 (47.6%)
Age class 66–80	3412 (14.9%)	1078 (24.1%)	4490 (16.4%)
Age class > 80	276 (1.2%)	81 (1.8%)	357 (1.3%)
Total	22,850 (100%)	4476 (100%)	27,326 (100%)

## Data Availability

The datasets presented in this article are not readily available because raw data on Hospital Discharge Records are routinely collected by law by the Ministry of Health, and are provided by the Ministry of Health—“Directorate-General for Health Planning—SDO database” to the Italian National Institute of Health for epidemiological purposes. Requests to access the datasets should be directed to the Italian Ministry of Health upon reasonable request. Restrictions apply to the availability of these data, which were used under license for the current study, and so are not publicly available. Derived data on aggregated form, used for statistical analysis and supporting the findings of this study, are available in Supplementary Materials.

## Supplementary Materials

The following supporting information can be downloaded at: https://www.mdpi.com/article/10.3390/audiolres15060175/s1, Figure S1: IRs for implanted cochlear and non-cochlear devices in Italy between 2001 and 2023 by age class. Table S1: Counts of implanted devices by sex, age class, and type of device. Years 2001–2023. Source: Hospital Discharge Records. Table S2: Incidence rates (×1,000,000 inhabitants) of implanted devices by sex, age class, and type of device. Years 2001–2023. Sources: Hospital Discharge Records; Italian Institute of Statistics. Table S3: Age-adjusted incidence rates (×1,000,000 inhabitants) of implanted devices by sex and type of device. Years 2001–2023. Sources: Hospital Discharge Records;Italian Institute of Statistics.

## Abbreviations

The following abbreviations are used in this manuscript:

SSD	Single-side deafness
HL	Hearing loss
CI	Cochlear implant
HDR	Hospital discharge record
ISS	Istituto Superiore di Sanità—Italian National Institute of Health
ICD9-CM	International classification of diseases—clinical modification
CI_95%_	95% confidence interval
IR	Incidence rate
AAIR	Age-adjusted incidence rate
IRR	Incidence rate ratio
