# Supplementary figures and images for: Trends and Incidence of Hearing Implant Utilization in Italy: A Population-Based Study
Source: Audiol Res. 2025 Dec 14;15(6):175. doi: 10.3390/audiolres15060175 (PMC12730023; doi:10.3390/audiolres15060175)

Age &lt; 1

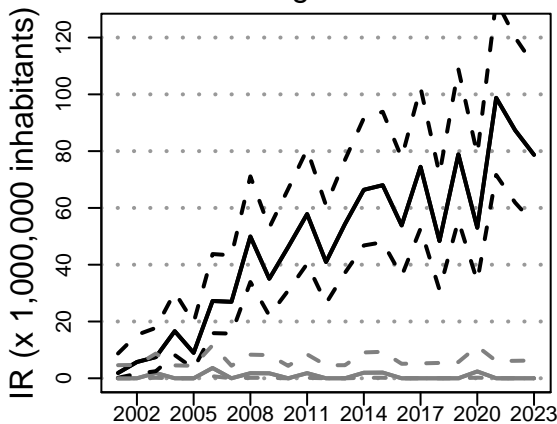

Age class 1–2

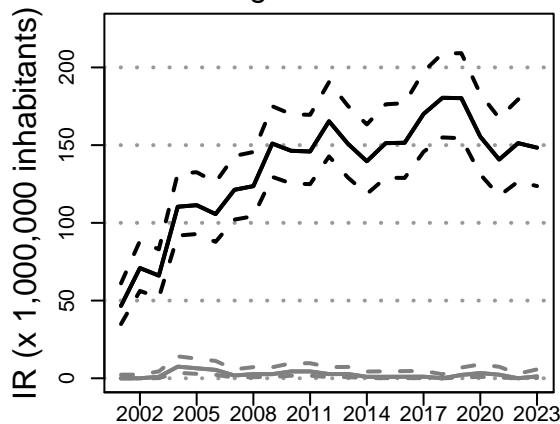

Age class 3–17

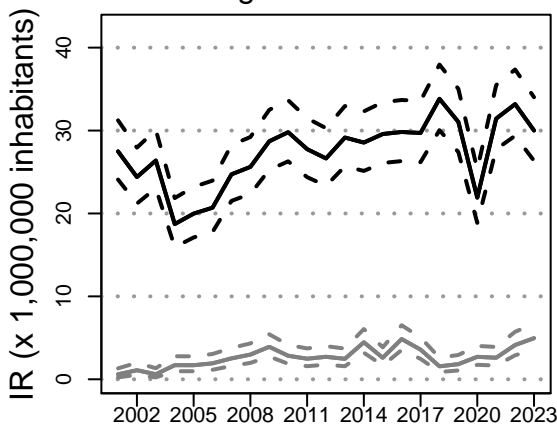

Age class 18–65

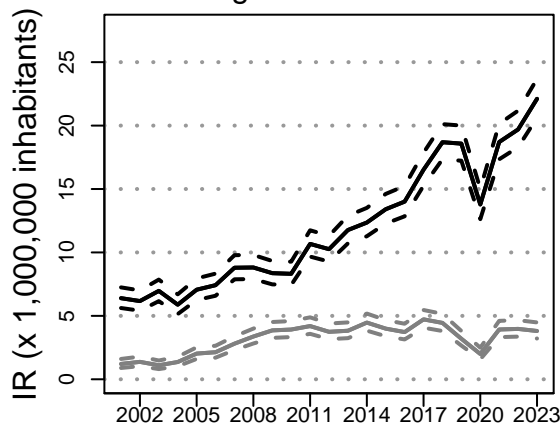

Age class 66–80

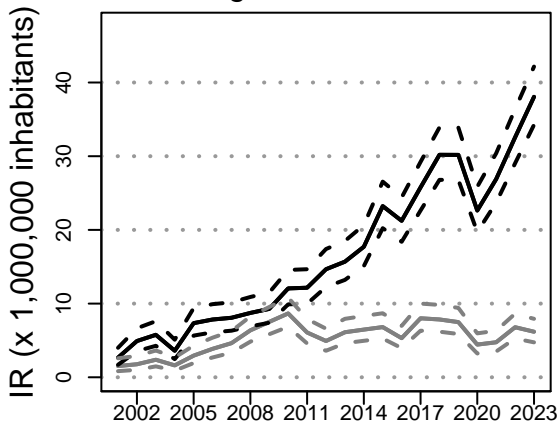

Age &gt; 80

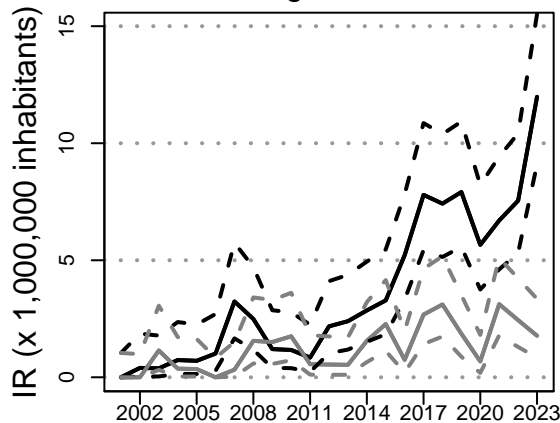

— IR

- - CI<sub>95%</sub>

■ Cochlear

■ Non-cochlear

Supplement: Supplementary file 1 [file audiolres-15-00175-s001.zip › FigureS1.pdf]
